# Genomic Detection of Multidrug‐Resistant H58 ST1 *Salmonella* Typhi in a South African Urban Waterway Reveals Epidemiological Links With Clinical Isolates

**DOI:** 10.1002/mbo3.70408

**Published:** 2026-09-27

**Authors:** Sanelisiwe Thinasonke Duze, Musa Marimani, Mrudula Patel

**Affiliations:** ^1^ Department of Clinical Microbiology and Infectious Diseases, National Health Laboratory Service and Faculty of Health Sciences University of Witwatersrand Johannesburg South Africa; ^2^ Department of Anatomical Pathology, Faculty of Health Science University of Witwatersrand Johannesburg South Africa

**Keywords:** biocide resistance, Jukskei River, multidrug resistance, *Salmonella* Typhi, whole genome sequencing

## Abstract

Typhoid fever remains a major public health concern in South Africa. However, the role of urban waterways as environmental reservoirs and contributors to the spread of resistant *Salmonella* Typhi remains poorly understood. This study characterized four *S*. Typhi isolates recovered from the Jukskei River in Johannesburg, South Africa, using whole‐genome sequencing. Genomic DNA was extracted and sequenced on the Illumina NextSeq. 2000 platform, and assembled genomes were analyzed for sequence types (STs), AMR determinants, plasmids, virulence factors, and phylogenetic relatedness using 124 clinical *Salmonella* genomes for comparison. All isolates were confirmed as *S*. Typhi, comprising ST1 (*n* = *2*) and ST2 (*n* = *2*). The ST1 isolates belonged to the H58 lineage (genotype 4.3.1.1), with isolate BRSA001 further classified as a sublineage 4.3.1.1. EA1. The ST1 isolates were multidrug‐resistant, carrying the IncQ1 plasmid and resistant genes, including *aph(6)‐Id*, *aph(3″)‐Ib*, *bla*_*TEM‐1B*_, *catA1*, *sul1*, *sul2*, and *dfrA7*. All isolates possessed mercury resistance genes (*merR*, *merT*, *merP*, *merC*), while ST1 isolates additionally harbored *qacEΔ1*. Ten *Salmonella* Pathogenicity Islands (SPI‐1 to SPI‐10) were identified in ST1 isolates. Phylogenetic analysis revealed notable genetic diversity among the river isolates. All river isolates clustered tightly (≤ 5 allele differences) with at least two clinical isolates, indicating shared epidemiological links and possible localized transmission. This study provides the first report of multidrug‐resistant H58 *S*. Typhi carrying *qacEΔ1* in South African surface water and highlights the Jukskei River as a potential environmental source of resistant *S*. Typhi, reinforcing the need for strengthened One Health genomic surveillance.

## Graphical abstract

Genomic analysis of four *S*. Typhi from a South African urban river uncovered two multidrug‐resistant H58 strains carrying *qacEΔ1*, revealing strong phylogenetic links to clinical isolates and underscoring urban rivers as potential transmission pathways.

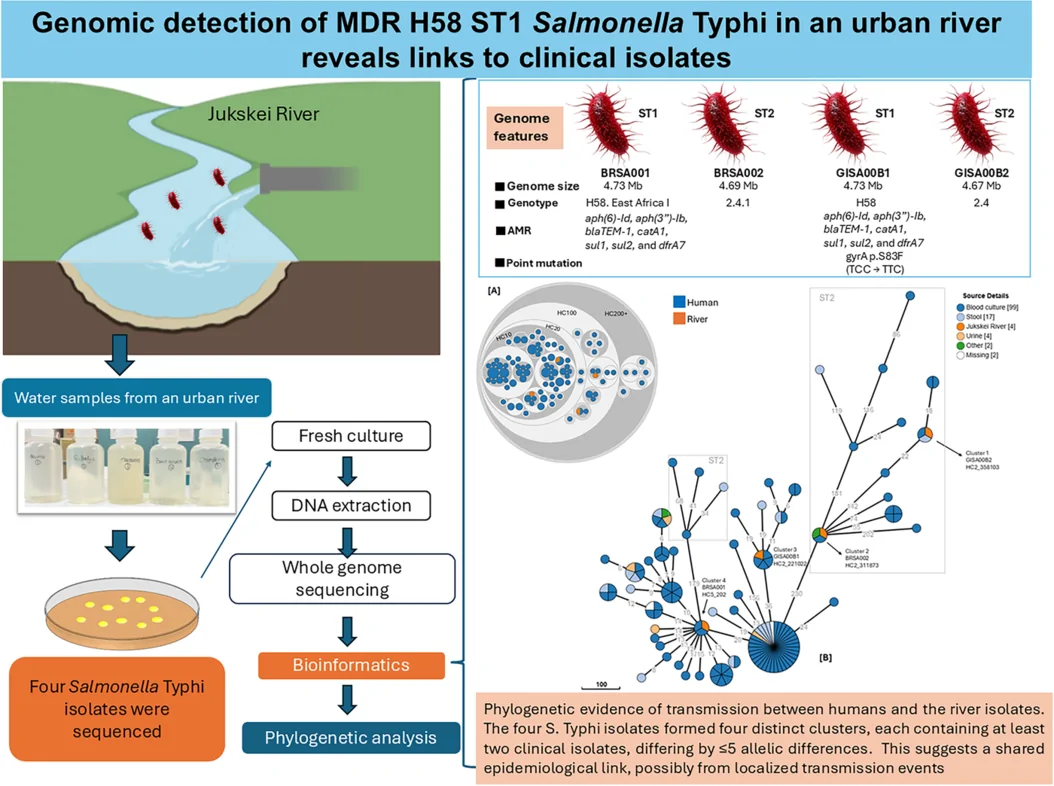

## 1 Introduction

Typhoid fever is a life‐threatening systemic infection caused by a Gram‐negative bacterium, *Salmonella enterica* subspecies *enterica* serovar Typhi, abbreviated to *Salmonella* Typhi (Meiring et al. 2023). Clinically, it presents with gastrointestinal symptoms such as diarrhoea and abdominal cramps, along with severe febrile systemic illness requiring prompt antibiotic treatment to prevent complications (Crump et al. 2015). Globally, typhoid fever accounts for more than 10 million illnesses annually and is associated with 100,000 deaths. Without effective antimicrobial therapy, the risk of severe complications and mortality increases substantially (GBD 2017 Typhoid and Paratyphoid Collaborators 2019 Typhoid and Paratyphoid Collaborators 2019). The vast majority of typhoid cases occur in low‐ and middle‐income countries (LMICs), where inadequate sanitation systems, contaminated water, and poor hygiene practices enable sustained transmission (Liu et al. 2025; Zhang et al. 2025).

Recent multicentre surveillance studies have highlighted typhoid fever as a significant health challenge across both urban and rural settings in sub‐Saharan Africa (Carey and Steele 2019; Marks et al. 2017). In South Africa, the disease is notifiable and continues to pose an important public health concern. Although the country is considered low endemicity, outbreaks still occur, particularly among vulnerable populations residing in informal settlements or densely populated areas with limited access to safe water and adequate sanitation (Keddy et al. 2018). One of the largest documented typhoid outbreaks in South Africa occurred in Delmas, Mpumalanga, in 1993, with over 1000 reported cases. While epidemiological investigations linked the outbreak to the municipal water supply, *S*. Typhi was not isolated from the collected water samples (Waner et al. 1998). Currently, outbreaks and sporadic cases in South Africa are identified through active laboratory‐based surveillance conducted by the Group for Enteric, Respiratory and Meningeal Disease Surveillance in South Africa (GERMS‐SA) at the National Institute for Communicable Diseases (NICD). Through active surveillance efforts supported by whole‐genome sequencing (WGS), five confirmed typhoid fever outbreaks have been detected in the past 5 years (Smith et al. 2025; Sekwadi et al. 2024). The most recent outbreak, which occurred between January and October 2025, resulted in 147 laboratory‐confirmed cases across eight provinces, with the highest burden in Gauteng (National Institute for Communicable Diseases 2025). To date, no definitive source has been identified for any of these outbreak‐associated cases (Smith et al. 2023). This persistent lack of a definitive transmission source highlights a critical blind spot in current surveillance efforts, which are largely centered on clinical case detection, with minimal systematic monitoring of environmental reservoirs.

Environmental surveillance of wastewater and surface waters provides an opportunity to monitor pathogen circulation beyond clinically diagnosed cases. Because *S*. Typhi is a human‐restricted pathogen shed in feces, wastewater networks and urban rivers may serve as sensitive epidemiological indicators of community‐level transmission (Matrajt et al. 2020). Specifically, environmental surveillance can be used to track disease prevalence, identify potential transmission pathways, and evaluate the effectiveness of interventions, including vaccination campaigns and improvements in water, sanitation, and hygiene infrastructure (Matrajt et al. 2020; Uzzell et al. 2023). This approach has been successfully implemented in diverse settings, including urban river systems and informal wastewater networks in Blantyre, Malawi, and high‐burden communities in Vellore, India, where environmental monitoring detected *S*. Typhi circulation independent of fluctuations in clinical case reporting (Uzzell et al. 2024; Abraham et al. 2025). Comparable data from South Africa remain scarce, underscoring the need to investigate environmental waters as potential indicators of typhoid transmission. As part of a broader study examining the occurrence of *Salmonella* spp. in an urban river in Johannesburg, Gauteng Province, South Africa (Duze et al. 2025), four *S*. Typhi isolates were recovered and subsequently characterized using WGS. Therefore, this study aimed to characterize the genomic features of these environmental *S*. Typhi isolates and assess their genetic relatedness to clinical isolates, thereby contributing to our understanding of typhoid circulation and the potential value of environmental surveillance in South Africa.

## 2 Materials and Methods

### 2.1 Bacterial Isolates

Water samples were collected in 2023 from the Jukskei River, a major urban waterway in Johannesburg, Gauteng Province, South Africa, as part of a broader longitudinal surveillance study previously reported by Duze et al. (2025). Briefly, sampling was conducted monthly over a 12‐month period at five sites along the river, yielding a total of 60 water samples. Four *S*. Typhi isolates were recovered during the study period, corresponding to a recovery rate of 6.7%. All four isolates were recovered in March 2023 from two sampling sites located approximately 3.35 km apart: Site A, located within a business district (26.18147° S, 28.10237° E), and Site B, situated within a recreational park (26.16475° S, 28.13029° E). Detailed sampling procedures, site characteristics, and the sampling map have been described previously (Duze et al. 2025).

### 2.2 DNA Extraction

Genomic DNA was extracted from overnight cultures grown on 5% blood agar using the QIAamp DNA Mini Kit (Qiagen, GmbH, Germany). DNA quantity and purity were assessed using a NanoDrop 2000 spectrophotometer (Thermo Fisher Scientific, MA, USA). The DNA samples were sent to the Sequencing Core Facility at the NICD for sequencing.

### 2.3 Whole Genome Sequencing and Analysis

Libraries were prepared with Illumina DNA Prep and sequenced on an Illumina NextSeq. 2000 platform using a 2 × 150 bp paired‐end protocol at 100× coverage. Adapter sequences were automatically removed during FASTQ generation by the Illumina NextSeq instrument. Illumina paired‐end reads were analyzed using the JEKESA bioinformatics pipeline v1.0 (https://github.com/stanikae/jekesa), which incorporates multiple bioinformatics analysis tools (Kwenda et al. 2020), briefly described below with default parameters. Quality control and read filtering of raw paired‐end reads were performed using FastQC v0.11.9 (Andrews 2010) and TrimGalore v0.6.7 (Krueger 2012). Reads were screened for contaminating sequences using Kraken2 against the MiniKraken2_v2_8GB database (Wood et al. 2019; Johns Hopkins University n.d.). Species identification and closest reference detection were performed using BactInspector version 0.1.3 (Underwood 2019), and intraspecies contamination was assessed using ConFindr (Low et al. 2019). Reads were assembled using SPAdes v3.14.1 (Bankevich et al. 2012) and polished with Shovill v1.1.0 (Seemann 2019), followed by quality assessment with QUAST v5.2.0 (Gurevich et al. 2013). Annotation was performed using the NCBI Prokaryotic Genome Annotation Pipeline v6.10 (Tatusova et al. 2016). In addition, Proksee v1.2.0 (Grant et al. 2023) was used to visualize gene features, including coding sequences (CDS), tRNAs, rRNAs, and regions for clustered regularly interspaced short palindromic repeat (CRISPR).

Further genotyping was performed on Pathogen Watch (https://pathogen.watch/), which identified the haplotype 58 (H58) lineage (Argimón et al. 2021). The Centre for Genomic Epidemiology (CGE) pipeline (http://www.genomicepidemiology.org/services/) was used for *Salmonella* serovar prediction using SeqSero2 version 1.1.0 (http://denglab.info/SeqSero2) (Zhang et al. 2015) and SISTR version 1.1.2 (https://github.com/phac-nml/sistr_cmd) (Yoshida et al. 2016). Multilocus sequence typing (MLST) based on PubMLST typing schemes (https://pubmlst.org/) (Jolley et al. 2018), antimicrobial resistance determinants, plasmids, virulence factors, and *Salmonella* pathogenicity islands (SPI) were also identified using tools on the CGE platform (MLST v2.0, ResFinder v4.7.2, PlasmidFinder v2.1, and SPIFinder v2.0) with default parameters (Larsen et al. 2012; Camacho et al. 2009; Bortolaia et al. 2020; Carattoli et al. 2014; Roer et al. 2016; Hupfeld et al. 2021). Lastly, MobileElementFinder v1.0.2 was used to detect the presence of mobile genetic elements, including plasmids, insertion sequences, and transposons, in the genome sequences and their association with the antimicrobial‐resistant genes (Camacho et al. 2009; Carattoli et al. 2014; Bankevich et al. 2012; Carattoli et al. 2014).

### 2.4 Phylogenetic Analysis

Phylogenetic analysis was performed using the CSI Phylogeny 1.4 pipeline (https://cge.cbs.dtu.dk/services/CSIPhylogeny/) to compare the four *S*. Typhi genomes. A maximum‐likelihood single‐nucleotide polymorphism (SNP) tree was generated using default parameters: minimum SNP depth of 10, relative SNP depth of 10, minimum inter‐SNP distance of 10 bp, SNP quality ≥ 30, read mapping quality ≥ 25, and Z‐score ≥ 1.96 (Kaas et al. 2014). *Salmonella enterica* subsp. *enterica* serovar Typhimurium strain LT2 (accession AE006468.2) served as the reference genome. The resulting phylogenetic tree was visualized and annotated using Interactive Tree of Life (iTOL) (Letunic and Bork 2024).

In addition, raw sequencing data were submitted to EnteroBase v1.2.0 (http://enterobase.warwick.ac.uk/species/index/senterica) (Achtman et al. 2021), where they were quality‐checked and assembled. The sequence data were analyzed via multiple tools to provide information concerning *Salmonella* serovar, AMR determinants, MLST, and core genome MLST (cgMLST). For phylogenetic analysis, all *S*. Typhi from “South Africa” collected in “2023” were selected for analysis on the EnteroBase. This selection criterion was used to ensure temporal comparability, as the environmental isolates characterized in this study were recovered in 2023. The Hierarchical Clustering (HierCC) of cgMLST profiles, incorporating the “cgMLST V2 + HierCC V1” scheme, was performed, which analyzed 3002 core genes of *Salmonella*. The genetic relatedness of the isolates was visualized using a HierCC bubble plot, as well as with a GrapeTree‐generated minimum spanning tree (MSTree) using the “MSTree V2” algorithm (Zhou et al. 2018). The HierCC bubble plot assigns genomes to stable, nested population clusters at multiple allelic distance thresholds (HC0 to HC200+ ), thereby revealing both fine‑scale and broad‑scale genetic relatedness among isolates. On the GrapeTree‐generated MST, the clusters were detected using the steps outlined in Smith et al. (2025). Briefly, once a GrapeTree was produced, the settings/operators of the tool were set to “collapse branches” at a value of “5,” which resulted in isolates showing ≤ 5 allelic differences being collapsed together into a “cluster.” Our cluster definition was ≥ 3 isolates showing ≤ 5 allelic differences, as found by the above actions, following the cgMLST analysis and creation of a GrapeTree. We defined a cluster of isolates at a ≤ 5‐allele difference threshold to denote high genetic relatedness among isolates and identify cases likely associated with a common source (epidemiological link or transmission event).

## 3 Results

### 3.1 Genomic Features

The genomic characteristics of the four *S*. Typhi isolates are summarized in Table 1. The genome sizes ranged from 4.67 to 4.73 Mbp, and all isolates displayed a GC content of 52.2%, consistent with typical *S. enterica* genomes. The number of CDS varied slightly (4551−4606 CDS), containing between 67 and 70 tRNA genes. Ribosomal RNA (rRNA) genes ranged from five to six per genome. All isolates carried one CRISPR array, suggesting a shared capacity for adaptive immunity against foreign genetic elements (Table 1). All four isolates were confirmed as *S*. Typhi and classified within serogroup D1. MLST analysis assigned the isolates to sequence types ST1 (*n* = *2*) and ST2 (*n* = *2*). The two ST1 isolates belonged to the H58 lineage (genotype 4.3.1.1), with isolate BRSA001 further resolved as a sublineage 4.3.1.1. EA1 (genotype 4.3.1 lineage I sublineage East Africa I).

**Table 1:** Genomic features of the four *Salmonella enterica* serovars Typhi from the Jukskei River.

Genomic features	BRSA001	BRSA002	GISA00B1	GISA00B2
Genome size (bp)	4,730,647	4,687,389	4,731,182	4,672,877
GC content (%)	52%	52%	52%	52%
Genes (total)	4689	4636	4685	4683
CDS (total)	4606	4551	4603	4602
tRNAs	68	70	68	67
rRNAs	6	6	5	5
CRISPR	1	1	1	1
Sequence type	1	2	1	2
Serogroup	D1	D1	D1	D1
Genotype	a4.3.1.1. EA1	2.4.1	a4.3.1.1	2.4
Collection site	A	A	B	B

a H58 Genotype.

### 3.2 Antimicrobial Resistance, Virulence Factors, and Stress Response

The ST1 isolates (BRSA001 and GISA00B1) exhibited multidrug resistance genotypes harboring the *aph(6)‐Id*, *aph(3”)‐Ib*, *blaTEM‐1*, *catA1*, *sul1*, *sul2*, and *dfrA7* genes, which confer resistance to aminoglycosides, β‐lactam, phenicol, sulfonamide, and trimethoprim, respectively. In addition, a point mutation (TCC → TTC) in the quinolone/triclosan resistance‐determining region in *gyrA* S83F was identified in the GISA00B1 isolate, as well as a stress‐response gene, *qacEΔ1*, in isolates BRSA001 and GISA00B1 (Figure 1).

**Figure 1:**
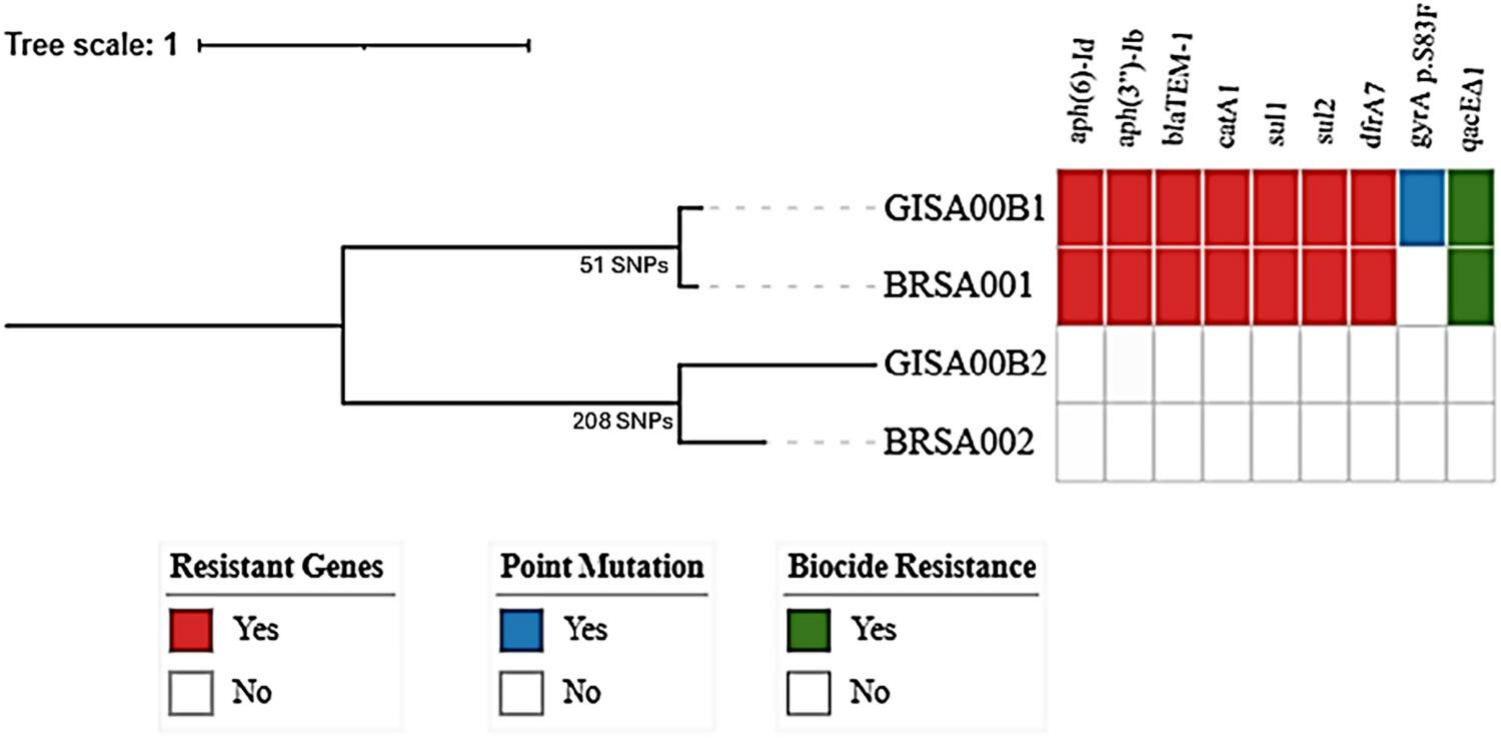
A SNP‐based maximum likelihood phylogenetic tree. The tree represents the phylogenetic relationships among the different isolates based on the SNP data obtained from the *S*. Typhimurium str. LT2 as a reference genome. The annotations on the tips of the tree provide information about each isolate, including resistant genes, point mutations, and biocide‐resistant genes detected. The tree is presented with branch lengths to emphasize relationships.

MobileElementFinder analysis identified the IncQ1 plasmid replicon and several insertion sequences, including IS26, ISSty2, MITEEc1, and ISSen6 (Table 2). In isolate BRSA001, the IncQ1 plasmid replicon was detected on contig00061 and was associated with the resistance genes *aph(6)‐Id*, *aph(3”)‐Ib*, and *sul2*. The *bla*_*TEM‐1B*_ gene was located on contig00066 together with the insertion sequence IS26.

**Table 2:** Associations between mobile genetic elements, antimicrobial resistance genes, and contigs identified in the multidrug‐resistant *S*. Typhi isolates BRSA001 and GISA00B1.

Isolate ID	MGE	ARGs	Contig
BRSA001	IncQ1	*aph(6)‐Id, aph(3”)‐Ib* and *sul2*	contig00061
	—	*catA1*	contig00060
	—	*qacEΔ1, sul1 and drfA7*	contig00063
	IS26	*bla*_*TEM‐1B*_	contig00066
	ISSty2	—	contig00003
	MITEEc1	—	contig00006
	ISSen6	—	contig00027
GISA00B1	IncQ1 and IS26	*aph(6)‐Id, aph(3”)‐Ib, sul2* and *bla*_*TEM‐1B*_	contig00052
	—	*qacEΔ1, sul1 and drfA7*	contig00057
	—	*catA1*	contig00059
	ISSty2	—	contig00004
	MITEEc1	—	contig00005
	ISSen6	—	contig00022

*Note:* no MGE or ARG detected.

In isolate GISA00B1, IncQ1 and IS26 were detected on contig00052, where they co‐occurred with *aph(6)‐Id*, *aph(3”)‐Ib*, *sul2*, and *bla*_*TEM‐1B*_ (Table 2). Notably, *qacEΔ1* was co‐localized with *sul1* and *dfrA7* on the same contig in both isolates (BRSA001: contig00063; GISA00B1: contig00057), although no plasmids or other mobile genetic elements were identified on these contigs. Similarly, the chloramphenicol resistance gene *catA1* was detected on separate contigs (BRSA001: contig00060; GISA00B1: contig00059) without association with identifiable MGE (Table 2).

The virulence profiles were uniform across all four isolates and included *sinH* (adhesion), iron acquisition genes (*iroB* and *iroC)*, and *cdtB* (toxin production). In addition, the presence of *SPI* (SPI‐1, SPI‐2, SPI‐3, SPI‐4, SPI‐5, SPI‐6, SPI‐7, SPI‐8, SPI‐9, and SPI‐10) were observed in the two ST1 isolates (BRSA001 and GISA00B1), while the ST2 isolates (BRSA002 and GISA00B2) exhibited a comparable SPI repertoire but lacked SPI‐4. Metal stress‐associated genes *merR*, *merT*, *merP*, and *merC*, which confer mercury resistance, were consistently detected in all four isolates.

### 3.3 Phylogenetic Analysis

The pairwise SNP analysis indicated that the four *S*. Typhi isolates were genetically distinct, differing by between 50 and 382 SNPs (Figure 1). Specifically, isolates GISA00B1 and BRSA001 differed by 51 SNPs and formed a sister‐taxon cluster in the phylogenetic tree, suggesting a recent shared ancestor. Whereas isolates GISA00B2 and BRSA002 differed by 208 SNPs and clustered together, indicating recent divergence. Collectively, these results suggest that the four isolates originated from different transmission sources (Figure 1).

An additional phylogenetic analysis using a total of 128 *S*. Typhi isolates (human, *n* = *124*, and Jukskei River, *n* = *4*) was performed on the EnteroBase Database using the cgMLST V2 + HierCC V1 scheme (Figure 2). The HierCC bubble plot revealed multiple nested clusters with different levels of genetic relatedness. The 124 *S*. Typhi isolates from clinical specimens belong to ST1 and ST2 STs and are distributed across the HierC clusters, with the majority of the isolates concentrated within the large HC10 cluster (Figure 2A). Although the four river isolates were collected in the same month from two sampling sites located 3.35 km apart, the isolates exhibited considerable diversity and clustered into four distinct phylogenetic clusters (Figure 2A,B). Each isolate formed a tight cluster with at least two clinical isolates within the smaller inner circles of ≤ 5 allele differences, resulting in four distinct clusters of closely related *S*. Typhi isolates (Figure 2A). The close clustering of environmental and clinical isolates suggests a potential epidemiological association.

**Figure 2:**
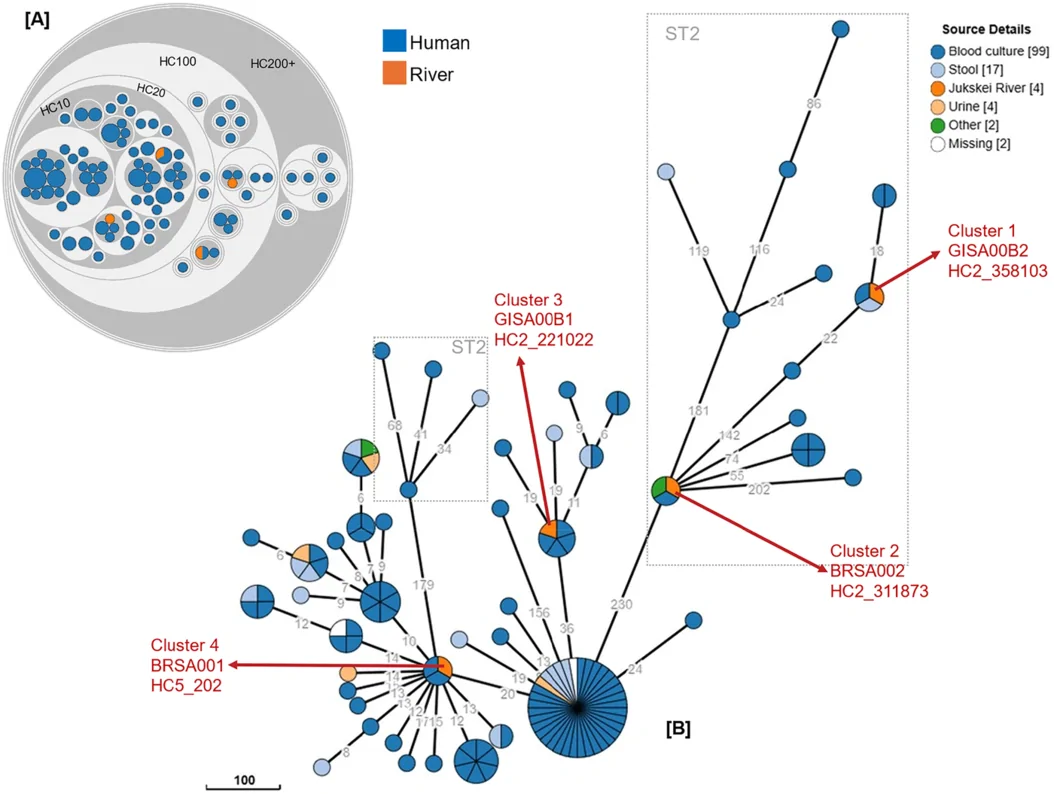
(A) Hierarchical clustering of *Salmonella* Typhi (*n* = *128*) from South Africa, 2023, based on cgMLST + HierCC scheme. Each circle represents a HierCC cluster at progressively increasing allelic thresholds (HC0 to HC200 + ), with smaller inner circles corresponding to fine‑scale, closely related clusters (HC0, HC2, and HC5) and larger outer rings representing broader population groupings (HC10, HC20, HC100, and HC200+). (B) Minimum spanning tree created based on cgMLST + HierCC scheme for *Salmonella* Typhi (*n* = *128*) from South Africa, 2023, by source detail. The spherical nodes represent isolates. Isolates showing ≤ 5 allelic differences were collapsed together into a single spherical node. The larger the spherical node, the more isolates it indicates. The number of segments within a spherical node is representative of the number of isolates. The numbers between adjoining nodes indicate the number of allele differences between the connecting nodes (isolates). A cluster was defined as ≥ 3 isolates showing ≤ 5 allelic differences. The gray boxes represent all ST2 isolates, with the isolates outside the gray boxes belonging to ST1.

The MSTree identified 12 clusters of *S*. Typhi, each comprising at least three isolates with ≤ 5 allele differences (Figure 2B). Four of these clusters contained *S*. Typhi isolates recovered from the Jukskei River, with cluster 3 being the largest, consisting of four clinical isolates and one environmental isolate from the river, all of which exhibited ≤ 2 allele differences (Figure 2B and Table 3). Clusters 1 and 2 were composed of ST2 *S*. Typhi, while Clusters 3 and 4 were mainly driven by ST1 *S*. Typhi H58 isolates (Figure 2B; Table 3). The clinical isolates within the four clusters were collected between February and April 2023, whereas the environmental isolates were recovered in March 2023. This temporal overlap suggests the co‐circulation of closely related *S*. Typhi lineages in environmental and clinical settings during the study period.

**Table 3:** Small *S*. Typhi transmission events that may have occurred between February and April 2023 based on the cgMLST + HierC scheme using the 128 isolates from clinical and the Jukskei River.

Cluster	River isolate	MLST	cgMLST	HierCC number^a^	Number of isolates^b^	Collection month^c^
1	GISA00B2	ST2	ST688335	HC2_358103	3	February
2	BRSA002	ST2	ST688338	HC2_311873	3	February
3	GISA00B1	ST1	ST688337	HC2_221022	5	April
4	BRSA001	ST1	ST688336	HC5_202	3	February

a HierCC—number of allele differences between the isolates in each cluster (i.e., HC2 means there are two allele differences between isolates).

b Number of isolates in the cluster.

c Collection month for the clinical isolates.

## 4 Discussion

*Salmonella* Typhi is a human‐restricted pathogen; therefore, its detection in the Jukskei River suggests faecal contamination of this urban waterway, likely arising from sewage discharge or poor sanitation infrastructure. In this study, four *S*. Typhi isolates recovered from the Jukskei River in March 2023 were characterized using WGS. These isolates belonged to two STs, ST1 and ST2, both of which represent the most commonly reported STs globally and which are frequently associated with *S*. Typhi in endemic settings (Stanaway et al. 2019; Díaz‐Torres et al. 2020; Fatima et al. 2023).

The ST1 *S*. Typhi isolates were further assigned to genotype 4.3.1, known as H58, based on SNP‐defined genotyping, with isolate BRSA001 further resolved as sublineage 4.3.1.1. EA1. The genotype 4.3.1 comprises three major lineages 4.3.1.1, 4.3.1.2, and 4.3.1.3 (Smith et al. 2023). The H58 lineage has been widely reported across sub‐Saharan Africa and Southeast Asia (Maes et al. 2020; Pragasam et al. 2020) and is the dominant genotype circulating in many parts of Southeast and South Asia, as well as East Africa. Phylogeographic analyses have demonstrated that H58 emerged in Asia before spreading internationally through multiple intercontinental transmission events, including repeated introductions into Africa, followed by sustained intra‐African transmission that facilitated its establishment across the continent (Wong et al. 2015). Consequently, this genotype is now well established across several African countries, including Tanzania, Kenya, Malawi, Zambia, Zimbabwe, and South Africa (Wong et al. 2015; Park et al. 2018; Dyson and Holt 2021).

In South Africa specifically, H58 *S*. Typhi has been documented in clinical isolates responsible for three major enteric fever outbreaks between 2020 and 2021 (Smith et al. 2023). However, to date, there have been no published reports of WGS‐confirmed H58 *S*. Typhi recovered from environmental sources such as rivers. Therefore, the detection of H58 in the Jukskei River represents a significant and novel finding, expanding current understanding of its ecological distribution and raising important questions regarding environmental reservoirs and transmission pathways. The H58 lineage of *S*. Typhi is of particular concern because of its strong association with multidrug resistance. In South Africa, all isolates linked to typhoid clusters in the report by Smith et al. (2025) were H58 and exhibited MDR profiles. Similarly, a study by Muturi et al. (2024) reported that *S*. Typhi lineage 4.3.1.2. EA3 strains isolated from a typhoid patient carried MDR determinants conferring resistance to ampicillin, sulfamethoxazole–trimethoprim, and chloramphenicol. Consistent with this pattern, the two ST1 H58 *S*. Typhi isolates recovered in our study were also MDR, harboring multiple resistance genes including *aph(6)‐Id*, *aph(3”)‐Ib*, *bla*_*TEM‐1B*_, *catA1*, *sul1*, *sul2*, and *dfrA7*. A study by Abdullah et al. (2025) reported that extensively drug‐resistant typhoid strains resistant to multiple first‐line antibiotics are linked to environmental contamination, with urban areas like Karachi demonstrating high rates of waterborne transmission. Therefore, the presence of MDR *S*. Typhi isolates in the Jukskei River emphasizes the role of contaminated water in spreading antibiotic resistance.

Antibiotic resistance is a significant public health threat, and *Salmonella* is a leading cause of antibiotic‐resistant bacterial infections in humans (Fatima et al. 2023). MDR in *S*. Typhi is frequently conferred by self‐transmissible plasmids carrying a suite of resistance genes, including resistance determinants for chloramphenicol (*catA1* or *cmlA*), ampicillin (*bla*_TEM‐1D_, *bla*_OXA‐7_), and co‐trimoxazole (at least one *dfr*A gene and at least one *sul* gene) (Holt et al. 2011; McMillan et al. 2020). Notably, the two ST1 H58 *S*. Typhi isolates carried the IncQ plasmid. The detection of an IncQ1 plasmid in environmental *S*. Typhi isolates within the same contig carrying antimicrobial resistance determinants (*aph(6)‐Id, aph(3”)‐Ib, sul2,* and *bla*_*TEM‐1B*_) is noteworthy given the established role of IncQ1 plasmids in the dissemination of resistance genes. The IncQ1 elements are known to be highly mobilizable, with a broad host range, and have been reported to disseminate AMR genes like *bla*_*TEM‐1B*_ across diverse bacterial species, with surface waters recognized as hotspots for plasmid‐mediated horizontal gene transfer (Mina et al. 2024; Boralli et al. 2025). In our study, however, complete plasmid reconstruction and conjugation experiments were not performed; therefore, the contribution of this plasmid to horizontal gene transfer in the Jukskei River remains to be determined.

Likewise, the presence of the insertion sequence IS26 in the ST1 H58 *S*. Typhi isolates further underscores its likely role in mediating the mobility of ARGs. IS26 is widely recognized as a key driver of genomic plasticity, facilitating the capture, rearrangement, and mobilization of resistance determinants (Harmer and Hall 2024; Tang et al. 2024; Wang et al. 2024). In a study by Wang et al. (2024), IS26 was responsible for 63.1% of all ARG transfer events observed among plasmids. Similarly, Harmer and Hall (2024) alluded that IS26 can recruit a gene or group of genes into the mobile gene pool, supporting their continued dissemination to new locations by creating pseudo‐compound transposons that can be further mobilized by the insertion sequence.

An interesting finding in our study was the detection of the *qacEΔ1* gene in the two ST1 H58 *S*. Typhi isolates. The *qacEΔ1* gene is associated with reduced susceptibility to quaternary ammonium compounds (QACs), a widely used class of disinfectants in food processing facilities (Duze et al. 2021; Chen et al. 2023). Previous work has shown that AMR genes and disinfectant resistance determinants frequently co‐localize on the same mobile genetic elements, including plasmids, integrons, and gene cassettes (Arrieta‐Gisasola et al. 2025). Notably, in both the ST1 H58 *S*. Typhi isolates, *qacEΔ1* was co‐localized with *sul1* and *dfrA7*. The co‐occurrence of *qacEΔ1* with AMR genes has been documented in *Salmonella* Indiana (Han et al. 2025). Similarly, Ali and Mohamed (2020) reported that *qacEΔ1* is commonly linked to class 1 integrons in nontyphoidal *Salmonella* (NTS), further supporting the role of these mobile genetic elements in facilitating the concurrent dissemination of disinfectant resistance and AMR determinants. Although *qacEΔ1* was co‐localized with *sul1* and *dfrA7* on the same contigs in both the ST1 H58 *S*. Typhi isolates, no plasmids, insertion sequences, or transposons were identified in association with these genes, limiting our ability to determine the mechanisms underlying their dissemination. Furthermore, phenotypic susceptibility to QAC was not assessed in this study. Consequently, the implications of the *qacEΔ1* gene remain unknown and warrant further investigation.

Nevertheless, the presence of the *qacEΔ1* gene is well documented in NTS, particularly in isolates associated with agricultural and food‑production environments. The *qacEΔ1* gene was recently reported in clinical MDR *S*. Typhi isolates from pediatric patients (Riaz et al. 2025). However, no study to date has described this gene in *S*. Typhi isolated from urban rivers. Therefore, the detection of *qacEΔ1* in our river *S*. Typhi isolates is novel and highlights the potential role of urban waterways in disseminating disinfectant‑ and antibiotic‑resistance determinants.

An important finding of this study was the close phylogenetic relationship between the environmental and clinical isolates. Specifically, the four *S*. Typhi isolates recovered from the Jukskei River formed four distinct clusters with clinical isolates, differing by ≤ 5 alleles. The observed clustering suggests a potential epidemiological association between environmental and clinical *S*. Typhi strains, indicating the circulation of closely related lineages in both environmental and human‐associated cases during the study period. In many LMICs, contamination of surface water and municipal supplies is a well‐established driver of typhoid transmission, often involving MDR strains (Levantesi et al. 2012; Mina et al. 2024). In contrast, identifying environmental sources during typhoid outbreaks in South Africa has remained challenging, with several investigations concluding without confirmation of a definitive source (Smith et al. 2023). Our findings, therefore, provide plausible genomic evidence supporting epidemiological links between urban waterways and human infection in this setting, further supporting the implementation of environmental surveillance as a complementary tool to enhance *S*. Typhi surveillance efforts.

Notably, the 2020–2023 national enteric fever surveillance data indicate that no outbreaks were notified during our sampling period. According to the Enteric Fever Surveillance Report, national case numbers in 2023 returned to baseline levels, with 141 laboratory‐confirmed cases and no cluster‐driven outbreaks reported for the year (Sekwadi et al. 2024). Therefore, the close phylogenetic clustering between river and clinical isolates suggests localized transmission events. Chronic sewage contamination of the Jukskei River likely supports the environmental persistence and recirculation of *S*. Typhi, creating potential exposure pathways for communities along the riverbanks, including those engaging in religious practices along the river. The small cluster sizes observed (< 5 isolates per cluster) are consistent with localized community‐level transmission rather than widespread dissemination. Although genomic relatedness cannot resolve the directionality of the transmission events, our findings highlight the need for integrated environmental and clinical surveillance. Notably, the use of genomic surveillance in South Africa has already facilitated the rapid detection of clusters indicative of emerging outbreaks. Therefore, incorporating systematic environmental monitoring, particularly of urban waterways, into the national surveillance framework will offer crucial insights into contamination sources, enhance early outbreak detection, and support more precise epidemiological investigations and public health responses.

## 5 Conclusion

This study provides the first evidence of the globally disseminated MDR H58 *Salmonella enterica* serovar Typhi lineage carrying the *qacEΔ1* gene in South African surface water, with clear phylogenetic links to clinical strains. The presence of these high‐risk genotypes in an urban river underscores the potential role of polluted urban waterways as overlooked reservoirs contributing to the persistence of typhoid and antimicrobial resistance genes in urban settings. Building on national advances in genomic surveillance, our findings highlight the value of extending the current genomic surveillance to include routine environmental monitoring of high‐risk rivers to strengthen early detection of contamination events and support more targeted and timely public health interventions within a One Health framework.

## Author Contributions

**Sanelisiwe Thinasonke Duze:** conceptualization, investigation, funding acquisition, writing – original draft, methodology, writing – review and editing, formal analysis. **Musa Marimani:** writing – review and editing, methodology, supervision. **Mrudula Patel:** conceptualization, supervision, writing – review and editing.

## Ethics Statement

An ethical waiver (W‐CBP‐191004‐02) was approved by the Human Research Ethics Committee (Medical) of the University of the Witwatersrand.

## Conflicts of Interest

None declared.

## Data Availability

The data that support the findings of this study are openly available in NCBI at https://www.ncbi.nlm.nih.gov/bioproject/PRJNA1332730, reference number RJNA1332730. All genome sequences generated from this study have been deposited in the National Center for Biotechnology Information (NCBI) database under BioProject accession https://www.ncbi.nlm.nih.gov/bioproject/PRJNA1332730, and the sequencing data were uploaded to the public EnteroBase platform (http://enterobase.warwick.ac.uk/species/index/senterica) and are freely available to access at this platform.
